# High Prevalence of Human Parvovirus 4 Infection in HBV and HCV Infected Individuals in Shanghai

**DOI:** 10.1371/journal.pone.0029474

**Published:** 2012-01-03

**Authors:** Xuelian Yu, Jing Zhang, Liang Hong, Jiayu Wang, Zhengan Yuan, Xi Zhang, Reena Ghildyal

**Affiliations:** 1 Microbiology Laboratory, Hongkou District Center for Disease Control and Prevention, Shanghai, People's Republic of China; 2 Shanghai Municipal Center for Disease Control and Prevention, Hongkou District Center for Disease Control and Prevention, Shanghai, People's Republic of China; 3 Microbiology Laboratory, Hongkou District Center for Disease Control and Prevention, Shanghai, People's Republic of China; 4 Respiratory Virology Group, Centre for Research in Therapeutic Solutions, Faculty of Applied Science, University of Canberra, Canberra, Australia; University Hospital San Giovanni Battista di Torino, Italy

## Abstract

Human parvovirus 4 (PARV4) has been detected in blood and diverse tissues samples from HIV/AIDS patients who are injecting drug users. Although B19 virus, the best characterized human parvovirus, has been shown to co-infect patients with hepatitis B or hepatitis C virus (HBV, HCV) infection, the association of PARV4 with HBV or HCV infections is still unknown.

The aim of this study was to characterise the association of viruses belonging to PARV4 genotype 1 and 2 with chronic HBV and HCV infection in Shanghai.

Serum samples of healthy controls, HCV infected subjects and HBV infected subjects were retrieved from Shanghai Center for Disease Control and Prevention (SCDC) Sample Bank. Parvovirus-specific nested-PCR was performed and results confirmed by sequencing. Sequences were compared with reference sequences obtained from Genbank to derive phylogeny trees.

The frequency of parvovirus molecular detection was 16–22%, 33% and 41% in healthy controls, HCV infected and HBV infected subjects respectively, with PARV4 being the only parvovirus detected. HCV infected and HBV infected subjects had a significantly higher PARV4 prevalence than the healthy population. No statistical difference was found in PARV4 prevalence between HBV or HCV infected subjects. PARV4 sequence divergence within study groups was similar in healthy subjects, HBV or HCV infected subjects.

Our data clearly demonstrate that PARV4 infection is strongly associated with HCV and HBV infection in Shanghai but may not cause increased disease severity.

## Introduction

Parvovirus 4 (PARV4) is a DNA virus belonging to the *Parvoviridae* family, and was first identified in 2005 [1]. PARV4 consists of two genotypes sharing 95% nucleotide identity, with the original PARV4 being classified into genotype 1 and the newly discovered PARV5 into genotype 2. *Parvoviridae* also includes B19, the first described human parvovirus, which causes erythema infectiosum and the recently described human Bocavirus (HboV) which is associated with lower respiratory tract infections.

PARV4 DNA has been detected in samples of pooled plasma from various manufacturers [2], [3], [4], [5] in different countries. So far, PARV4 DNA has been restricted to subjects with a parenteral exposure history suggesting a predominantly or exclusively parenteral route of transmission [4], [6], [7]. In one study, PARV4 DNA has been detected in blood samples from cadavers of injecting drug users who were also HCV RNA positive [8]. Taken together with previous work showing that B19 is able to co-infect with HCV, this suggests that PARV4 might be able to co-infect with HCV with possible implications for disease severity.

In this study we have investigated the prevalence of PARV4 in healthy adults, HCV and HBV infected subjects, examined the predominant genotype of the virus causing PARV4 infection in Shanghai and its association with HCV and HBV infection.

## Methods

### Study Groups

In our study, we used 153 serum samples from HCV positive, 248 from HBV positive patients and 289 from healthy individuals matched for age and year of sample collection. Sera positive for HCV IgM antibody (Anti-HCV IgM) and negative for HBV S antigen (HBsAg) were termed HCV infected subjects (Numbered from HCV001-HCV153); sera negative for both Anti-HCV IgM and HBsAg collected in year of 2008 were chosen as controls for HCV-infected subjects (Numbered from H08001-H08096); sera positive for HBsAg, but negative for HBe antigen (HBeAg) and anti-HCV IgM were termed HBV infected subjects (Numbered from HBV001-HBV188); sera negative for both anti-HCV IgM and HBsAg collected in year of 2009 were chosen as controls for HBV infected subjects (Numbered from H09001-H09193). Sixty sera positive for both HBsAg and HBeAg detection were classed as Chronic HBV Infection. All samples were retrieved from Shanghai Centers for Disease Control and Prevention (SCDC) Sample Bank. All human sera were de-identified before use in the study and all laboratory testing with samples was approved by SCDC Ethical Review Committee. All work involving human samples was approved by the Ethical Review Committee, Shanghai Municipal Center for Disease Control and Prevention. The samples were collected as part of routine surveillance activities undertaken by SCDC, and consent waived by the Ethical Review Committee.

### Serological ELISA Assays

Anti-HCV IgM and HBsAg were assayed in all serum samples used. HBeAg was assayed in sera positive for HBsAg. Anti-HCV IgM, HBsAg and HBeAg were assayed using commercial ELISA kits (Diagnostic Kit for Anti-HCV IgM, HBsAg and HBeAg from Kehua Cooperation, Shanghai). Experiments were performed as per manufacturer's recommendations. Positive and negative controls were included in each test. Any sample with OD of sample/average OD of negative control ≥2.1, was taken as positive.

### PARV4 PCR

The total DNA of each sera sample (200 µl) was isolated on the MagNA Pure LC 2.0 (Roche, Switzerland) using MagNA Pure LC DNA Isolation Kit (Roche, Germany) following manufacturer's recommendations; 60 µl of eluted DNA was used as template for PCR.

PARV4 nested PCR was performed in all samples using previously described primers PV4ORF1F and PV4ORF1R (outer primers) [3] and PV4NS1Fn2 and PV4NS1Rn2 (inner primers) [5], with GoTaq PCR Core System II (Promega, USA). PCR reaction conditions were as previously published [5].

### B19 and Bocavirus Real-time PCR

Human Parvovirus (B19) Real Time PCR kit and HBoV Real Time PCR kit (Both from Shanghai ZJ Bio-Tech Co., Ltd., People's Republic of China) were used to perform B19 and HBoV real-time PCR respectively, in all sera.

### HBV DNA Test and ALT Assay

Serum specimens of chronic HBV patients were tested for presence of viral DNA with a Real Time PCR Quantitation kit (ZJ Bio-Tech, Shanghai). The PCR mix was amplified by Roche Light Cycler 480 (Roche, Switzerland). Alanine aminotransferase (ALT) was assayed in sera from chronic HBV patients using commercial kits (Kehua Cooperation, Shanghai) detected by the Synchron CX® 5 PRO Clinical Systems (Beckman, USA). Experiments were performed as per instructions; positive and negative controls provided by the company were included in each test. Serological positivity was accepted at titers ≥64 units per liter (U/L).

### Sequencing and Phylogenetic Analysis

Identity of nested PCR products was confirmed by sequencing. PCR amplicons from 1564 to 1724 bp of ORF1 were sequenced by Biosune Company using the internal primers (BioSune, Shanghai). Sequences were analyzed by CLC Sequence Viewer 6 [9]. To facilitate phylogenetic analysis, PARV4 (PARV4 genotype 1; AY622943.1 and EU546211.1) and PARV5 (PARV4 genotype 2) sequences (DQ112361.1 and DQ873391.1) were downloaded from GenBank.

### Data Analysis

Data were processed by SPSS for Windows version 13.0. Differences between nominal or ordinal variables were tested by the Pearson's Chi-Square test. Continuous numeric data were compared by t test or nonparametric Kruskal-Wallis Chi-Square test. Significance was accepted at *p*≤0.05.

## Results

### Study subjects

The general information regarding study subjects is listed in Table 1. Groups were compared for gender and age distribution. 65.4% (100/153) of the HCV infected subjects and 67.6% (65/96) of matched controls were male (p = 0.703). Similarly, there was no difference in the gender distribution between HBV infected subjects and matched controls (p = 0.590). HCV infected subjects had the median age of 44.5 years, which was not different from that of matched controls (41 years; p = 0.227). The median age of HBV infected subjects was 41 years and did not differ from that of controls (36 years; p = 0.221).

**Table 1:** General information of study subjects.

Characteristic	Healthy Controls	Patients	p Value
**Gender**			
HCV group			
Male	67.6% (65/96)	65.4% (100/153)	0.703
Female	32.3% (31/96)	34.6% (53/153)	
HBV group			
Male	57.0% (110/193)	54.3% (102/188)	0.590
Female	43.0% (83/193)	45.7% (86/188)	
**Age (years)**			
HCV group			
Median	41	44.5	0.227
IQR	35–47	37–49	
Range	16–68	21–57	
HBV group			
Median	36	41	0.221
IQR	26–46	28–47.5	
Range	18–62	17–60	

### B19 and Bocavirus Real-time PCR

Neither B19 nor HBoV were detected in serum samples from HBV, HCV infected subjects or their corresponding healthy controls.

### HBV or HCV infected subjects had increased incidence of PARV4 DNA in sera compared to matched controls

Sera from 33.3% (51/153) of HCV infected subjects but only 16.7% (16/96) of matched controls were positive for PARV4 DNA (p = 0.004, Table 2). There was no difference in the gender distribution (p = 0.336) between HCV infected subjects who were positive for PARV4 (70.6%; 36/51 male) compared to those who were negative (62.7%; 64/102 male). Similarly, compared with the matched control group, PARV4 DNA detection was significantly higher in HBV infected subjects (p<0.0001), with no difference in the gender distribution (p = 0.122) between HBV infected subjects positive (60.3%; 47/78 male) or negative (50%; 55/110 male) for PARV4 (Table 2). Further analysis of PARV4 prevalence among different groups suggested that frequency of positive PARV4 DNA test was not different between HCV infected subjects and HBV infected subjects (p = 0.079), or between HCV matched controls from 2008 and HBV matched controls from 2009 (p = 0.283).

**Table 2:** Detection Rate of PARV4 DNA.

Characteristic	PARV4 Positive	PARV Negative	p Value
**Rate Between Patients and Healthy Controls**			
HCV group			
Controls	16.7% (16/96)	83.3% (80/96)	0.004
Patients	33.3% (51/153)	66.7% (102/153)	
HBV group			
Controls	22.2% (43/193)	77.7% (150/193)	<0.0001
Patients	41.5% (78/188)	58.5% (110/188)	
**Rate Between Genders**			
HCV group			
Male	70.6% (36/51)	62.7% (64/102)	0.336
Female	29.4% (15/51)	37.3% (38/102)	
HBV group			
Male	60.3% (47/78)	50.0% (55/110)	0.122
Female	39.7% (31/78)	50.0% (55/110)	
**Rate Between HBV and HCV, as well as between Healthy Controls**			
Hepatitis Patients			
HCV	33.3% (51/153)	66.7% (102/153)	0.079
HBV	41.5% (78/188)	58.5% (110/188)	
Healthy Controls			
2008	16.7% (16/96)	83.3% (80/96)	0.283
2009	22.2% (43/193)	77.7% (150/193)	

### PARV4 Infection is not associated with severity of HBV disease

Statistical analysis showed no difference in frequency of PARV4 DNA detection between the HBV infected (HBV carriers) and chronic HBV patients (p = 0.665), see Table 3. Further statistical analyses within the 60 chronic HBV patients showed similar frequency of PARV4 DNA detection between HBV DNA positive and negative patients (p = 0.597) or ALT positive (ALT≥64 U/L) and negative (ALT<64 U/L) patients (p = 0.500). PARV4 positive subjects had an average ALT expression of 94.04 U/L, which was not significantly different from that of PARV4 negative subjects (107.3 U/L, p = 0.687; see Table 4).

**Table 3:** General information & PARV4 DNA positive rate of HBV infected groups.

Characteristic	HBV Infection (HBsAg^+^ HBeAg^−^)	Chronic HBV Infection (HBsAg^+^ HBeAg^+^)	p Value*
**Gender**			
Male	57.0%(107/188)	56.7%(34/60)	0.744
Female	43.0%(81/188)	43.3%(26/60)	
**Age**			
Median	41	42	0.245
IQR	28–47.5	36–48	
**PARV4 DNA**			
Positive	41.5%(78/188)	38.3%(23/60)	0.665
Negative	58.5%(110/188)	61.7%(37/60)	

*p value: data for 2 HBV patient groups was compared with Pearson's χ2- test.

**Table 4:** The PARV4 nucleic acid frequency in chronic HBV patients with/without HBV DNA, and with different ALT levels.

Characteristic	PARV Positive	PARV Negative	P Value
**HBV DNA**			
Positive	60.9% (14/23)	39.1% (9/23)	0.597
Negative	67.6% (25/37)	32.4% (12/37)	
**ALT Quantitative**			
Mean of ALT (U/L)	94.04	107.30	0.687
**ALT Qualitative**			
Positive (ALT≥64 U/L)	52.2% (12/23)	43.2% (16/37)	0.500
Negative (ALT<64 U/L)	47.8% (11/23)	56.8% (21/37)	

### High Sequence Similarity in PARV infecting Healthy, HBV or HCV infected subjects

**Figure 1:**
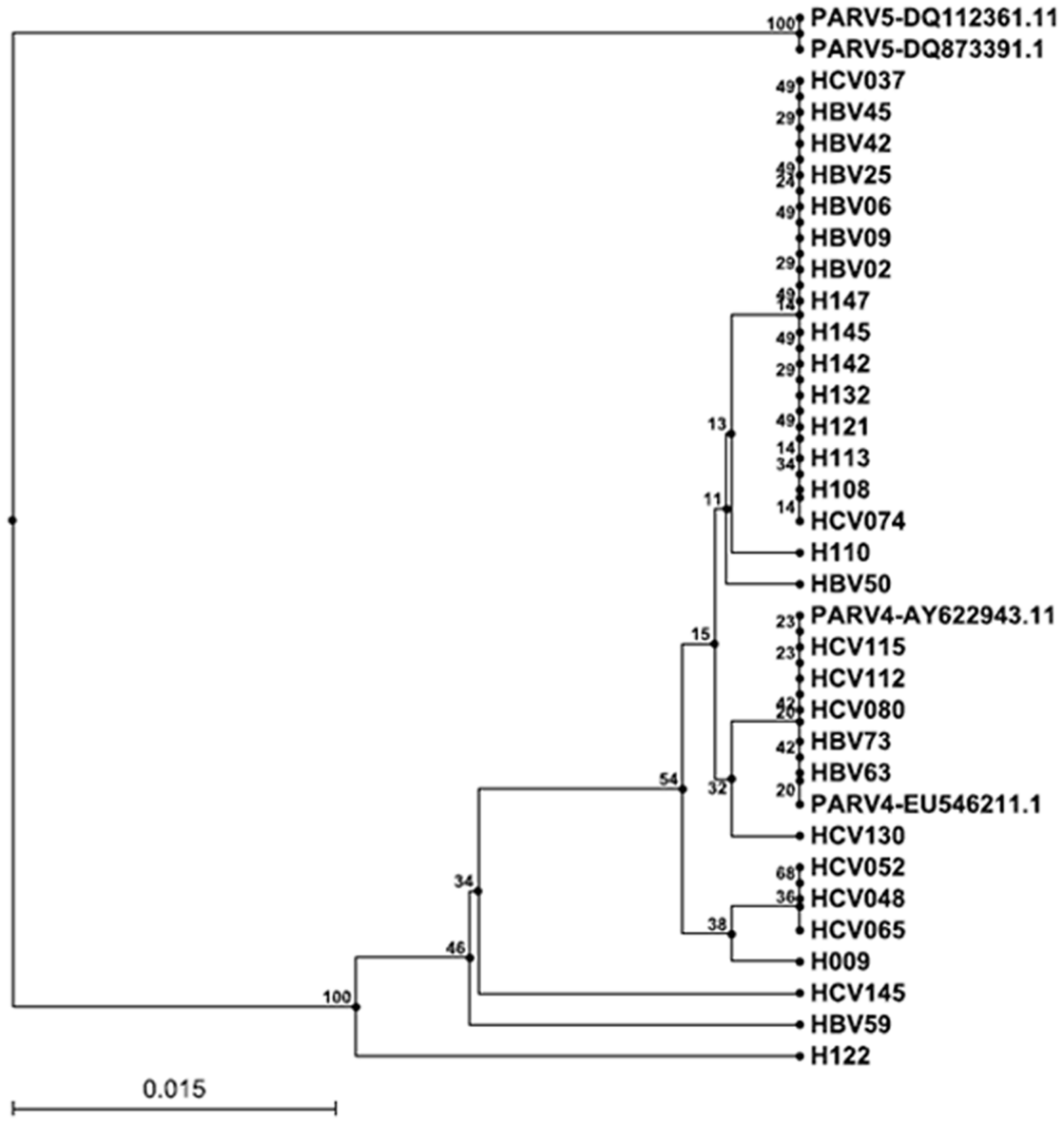
Phylogenetic tree of PARV4 isolates infecting HBV, HCV patients and healthy controls. The nucleotide sequence from 1564 to 1724 bp of ORF1 gene of PARV4 were amplified, sequenced and analyzed. Bootstrap percentages are given at the node of each branch (1000 replicates). Sequences H009, H108, H110, H113, H121, H122, H132, H142, H145 and H147 were from healthy controls. Sequences HBV02, HBV06, HBV09, HBV25, HBV42, HBV45, HBV50, HBV59, HBV63 and HBV73 were from HBV infected subjects with PARV4 infection. Sequences HCV037, HCV048,HCV052, HCV065, HCV074, HCV080, HCV112, HCV115, HCV130 and HCV145 were from HCV infected subjects with PARV4 infection. AY622943.1 and EU546211.1 represent 2 PARV4 genotype 1 reference strains, and DQ112361.11 and DQ873391.1 are sequences of 2 PARV4, genotype 2 reference strains. All reference strain sequences were downloaded from GenBank.

Ten sequences were selected from each study group and used to construct an evolutionary tree (Fig. 1). All 30 study subjects (Genbank accession number range from HQ267348 to HQ267375) were infected with PARV4, with nucleotide sequence homology ranging from 93.8% to 100%. The sequence homology between study samples and PARV4 genotype 1 reference strains ranged from 95.7 to 99.5%; between study samples and PARV4 genotype 2 reference strains from 90.1–93.6%. PARV4 detected in sample H122 showed the highest variability among the study samples (93.8–95.7%), with 95.7% homology with PARV4 genotype 1 reference sequences, and 90.1% with PARV4 genotype 2 reference sequences. The genetic distances between PARV4 and HBV59, HCV145 and H009 sequences were slightly larger than that of other study subjects. When an evolutionary tree was constructed on the basis of year of collection of samples no change in strains was observed in successive years.

## Discussion

Previous studies have suggested a significant correlation between HBV and B19 co-infection [10], [11]. PARV4 was originally detected in a HBV infected patient raising the possibility of an association with HBV infection as has been described for B19 [1], [12]. To determine the association of PARV4 infection with HBV and HCV infection, we investigated the molecular prevalence of PARV4 in healthy individuals, HBV-infected subjects and HCV-infected subjects in Shanghai.

The frequency of PARV4 DNA detection was significantly higher among HBV infected and HCV infected subjects than matched controls. Our data demonstrate that PARV4 is probably able to co-infect with HBV and HCV. However, co-infection with PARV4 and HBV may not increase the severity of liver dysfunction in chronic HBV patients as no correlation between PARV4 prevalence and HBV DNA expression, or between PARV4 and blood ALT was observed. Our results show a similar trend to a study from Scotland which did not find any correlation between immunosuppression and PARV4 viral load or between PARV4 viral load and CD4 lymphocyte counts or HIV viral loads in HIV/AIDS patients [13]. 16.7–22.2% of healthy individuals in Shanghai had contracted PARV4 without any known acute viral infection symptoms, suggesting that PARV4 infection may often cause only subclinical infection.

The frequency of detection of PARV4 in sera from healthy subjects (16.7–22.2%) in our study is significantly higher than that reported in plasma from healthy blood donors from North America (5%, 7 of 137) [3] or Europe (none) [13]. Moreover, the PARV4 DNA prevalence in HCV and HBV infected individuals in Shanghai is markedly higher than that in highly exposed and symptomatic HIV patients from USA (33.3–41.5% vs. 6%) [12] or HCV-infected injecting drug users (IDUs) from London (30%; 3 of 10) [8] similar to that in plasma-derived coagulation factor concentrates from Germany (33%; 7/21) [14] and lower than that in the bone marrow and lymphoid organs of HIV-positive individuals (70%; 17 of 24) from Scotland [13]. Our data are in agreement with recent data from France where PARV4 DNA was detectable in 27.3% (6/22) and 38.7% (29/75) of plasma samples from haemodialysis patients positive for HCV and HBV, respectively [15]. The high prevalence of PARV4 DNA in HBV, HCV and HIV-infected subjects may simply reflect a generally higher exposure to infectious agents in these population groups. The variation in detection frequency in different countries may be multifactorial, such as the study sample size, sample types, the way and time of sample collection and storage, as well as detection protocols. In our study, the frequency of PARV4 detection in samples from 2009 was slightly higher than that in samples from 2008 (p = 0.283); probably reflecting the loss of quality due to longer storage period.

HIV-infected IDUs have a high prevalence of PARV4 infection, both by molecular detection of PARV4 infection and presence of PARV4 IgG antibody [6], [7], [16], which suggests that PARV4 may be transmitted through parenteral routes. We cannot form any conclusions about the possible transmission mode of PARV4 in our study with the limited information available. However, given the high prevalence of PARV4 DNA in sera of healthy subjects in Shanghai, blood or blood products were probably not the major route of transmission in our study group; the possibility that PARV4 may be transmitted via the parenteral route in some settings, similar to B19, cannot be dismissed [16].

In our study, the median age of study subjects with PARV4 infection was 33.5 (IQR, 25–47), ranging from 23 to 57 which is different from the reported age range of PARV4 infected individuals in Edinburgh where all positive samples were from subject born in the 1960s [13]. Our findings also suggest that PARV4 may cause persistent infection as the age of the oldest individual with PARV4 infection was nearly sixty. A recent study of T cell responses to PARV4 in HCV and HIV positive individuals suggests that PARV4 infections are common in these clinical groups and that viral antigen persists to trigger and maintain T cell responses [17].

Phylogenetic analysis showed that all sequences of PARV4 ORF1 detected in this study clustered together with the two PARV4 genotype 1 reference strains and had a more distant phylogenetic relationship with PARV4 genotype 2 (PARV5). PARV4 genotype 1 may be the only PARV4 causing epidemics in Shanghai. Interestingly, the PARV4 genotype 1 variants circulating in Shanghai were substantially more divergent than those in Edinburgh [13].

Our study found that PARV4 has been circulating in Shanghai for the past 50 years with up to 22% of the population being affected. PARV4 had a significantly higher prevalence in HBV infected and HCV infected compared to healthy subjects. The pathogenic mechanisms of PARV4 co-infecting with other persistent pathogens remains to be elucidated.

## References

[1] Jones MS, Kapoor A, Lukashov VV, Simmonds P, Hecht F (2005). New DNA viruses identified in patients with acute viral infection syndrome.. J Virol.

[2] Fryer JF, Hubbard AR, Baylis SA (2007). Human parvovirus PARV4 in clotting factor VIII concentrates.. Vox Sang.

[3] Fryer JF, Kapoor A, Minor PD, Delwart E, Baylis SA (2006). Novel parvovirus and related variant in human plasma.. Emerg Infect Dis.

[4] Lurcharchaiwong W, Chieochansin T, Payungporn S, Theamboonlers A, Poovorawan Y (2008). Parvovirus 4 (PARV4) in serum of intravenous drug users and blood donors.. Infection.

[5] Vallerini D, Barozzi P, Quadrelli C, Bosco R, Potenza L (2008). Parvoviruses in blood donors and transplant patients, Italy.. Emerg Infect Dis.

[6] Sharp CP, Lail A, Donfield S, Simmons R, Leen C (2009). High frequencies of exposure to the novel human parvovirus PARV4 in hemophiliacs and injection drug users, as detected by a serological assay for PARV4 antibodies.. J Infect Dis.

[7] Simmonds P, Manning A, Kenneil R, Carnie FW, Bell JE (2007). Parenteral transmission of the novel human parvovirus PARV4.. Emerg Infect Dis.

[8] Fryer JF, Lucas SB, Padley D, Baylis SA (2007). Parvoviruses PARV4/5 in hepatitis C virus-infected patient.. Emerg Infect Dis.

[9] Bateman A, Coin L, Durbin R, Finn RD, Hollich V (2004). ClC free workbench 3.0.2.. Nucleic Acids Res.

[10] Hsu TC, Chen TY, Lin MC, Tzang BS, Tsay GJ (2005). Human parvovirus B19 infection in patients with chronic hepatitis B or hepatitis C infection.. J Gastroenterol Hepatol.

[11] Toan NL, Song le H, Kremsner PG, Duy DN, Binh VQ (2006). Co-infection of human parvovirus B19 in Vietnamese patients with hepatitis B virus infection.. J Hepatol.

[12] Fryer JF, Delwart E, Hecht FM, Bernardin F, Jones MS (2007). Frequent detection of the parvoviruses, PARV4 and PARV5, in plasma from blood donors and symptomatic individuals.. Transfusion.

[13] Manning A, Willey SJ, Bell JE, Simmonds P (2007). Comparison of tissue distribution, persistence, and molecular epidemiology of parvovirus B19 and novel human parvoviruses PARV4 and human bocavirus.. J Infect Dis.

[14] Schneider B, Fryer JF, Oldenburg J, Brackmann HH, Baylis SA (2008). Frequency of contamination of coagulation factor concentrates with novel human parvovirus PARV4.. Haemophilia.

[15] Touinssi M, Reynaud-Gaubert M, Gomez C, Thomas P, Dussol B (2011). Parvovirus 4 in French in-patients: a study of hemodialysis and lung transplant cohorts.. J Med Virol.

[16] Heegaard ED, Petersen BL, Heilmann CJ, Hornsleth A (2002). Prevalence of parvovirus B19 and parvovirus V9 DNA and antibodies in paired bone marrow and serum samples from healthy individuals.. J Clin Microbiol.

[17] Simmons R, Sharp C, Sims S, Kloverpris H, Goulder P (2011). High frequency, sustained T cell responses to PARV4 suggest viral persistence in vivo.. J Infect Dis.

