# Correction: High Prevalence of Human Parvovirus 4 Infection in HBV and HCV Infected Individuals in Shanghai

**DOI:** 10.1371/annotation/c9dc0a19-a6b4-4b88-9305-660189544613

**Published:** 2012-11-13

**Authors:** Xuelian Yu, Jing Zhang, Liang Hong, Jiayu Wang, Zhengan Yuan, Xi Zhang, Reena Ghildyal

There was an error in affiliation 1 for authors Xuelian Yu, Jiayu Wang, Xi Zhang, as well as an error in affiliation 2 for author Zhengan Yuan. Affiliations 1 and 2 should be as follows: **1** Microbiology Laboratory, Shanghai Municipal Center for Disease Prevention and Control, Shanghai, People's Republic of China, **2** Shanghai Municipal Center for Disease Prevention and Control, Shanghai, People's Republic of China 

